# Noncoding RNAs as a novel approach to target retinopathy of prematurity

**DOI:** 10.3389/fphar.2022.1033341

**Published:** 2022-10-20

**Authors:** Hyunjong Kim, Jaesub Kim, Juhee Ryu

**Affiliations:** ^1^ Vessel-Organ Interaction Research Center, College of Pharmacy, Kyungpook National University, Daegu, South Korea; ^2^ College of Pharmacy and Research Institute of Pharmaceutical Sciences, Kyungpook National University, Daegu, South Korea

**Keywords:** microRNA, long noncoding RNA, circular RNA, retinopathy of prematurity, retinal vascular disease, noncoding RNA

## Abstract

Retinopathy of prematurity (ROP), a vascular disease characterized by abnormal vessel development in the retina, has become a primary cause of blindness in children around the world. ROP can be developed during two different phases: vessel loss and vessel proliferation. Once preterm infants with immature retinal vessel growth are exposed to high level of oxygen inside the incubator, vessel loss can occur. When infants are exposed to room air, they may experience the proliferation of vessels in the retina. Although multiple factors are reported to be involved in the pathogenesis of ROP, including vaso-endothelial growth factors (VEGFs) and hypoxia-inducible factors, the pathogenesis of ROP is not completely understood. Although laser therapy and pharmacologic agents, such as anti-VEGF agents, have been commonly used to treat ROP, the incidence of ROP is rapidly rising. Given that current therapies can be invasive and long-term effects are not fully known, the search for novel therapeutic targets with less destructive properties needs to be considered. Within the last decade, the field of noncoding RNA therapy has shown potential as next-generation therapy to treat diverse diseases. In this review, we introduce various noncoding RNAs regulating ROP and discuss their role as potential therapeutic targets in ROP.

## Introduction

### Retinopathy of prematurity

Retinopathy of prematurity (ROP) is a progressive retinal vascular disease that occurs in preterm infants. It is characterized by abnormal vessel growth in the retina. The incidence of ROP has gradually increased due to the development of neonatal care and has become a leading cause of childhood blindness (Freitas et al., 2018; Filippi et al., 2022). Various factors including oxygen supplementation, low birthweight or gestational age, blood transfusion, and sepsis can be risk factors for ROP (Alajbegovic-Halimic et al., 2015). The progression of the disease can be divided into two phases (Hartnett and Penn, 2012). During phase 1, retinal vessel development is delayed due to various factors, such as hyperoxia caused by oxygen supplementation, reactive oxygen stress, or low maternal-derived factors (Graziosi et al., 2020). As a result, a peripheral avascular region may occur in preterm infants. During phase 2, when the preterm infants are returned to room air, the proliferation of retinal vessels can occur in the avascular region of the retina causing vision loss (Hartnett and Penn, 2012).

Diverse molecular factors and signaling cascades are involved in the development of ROP (Hartnett, 2015; Ryu, 2022). There is evidence of downregulation of hypoxia-inducible factor 1-alpha (HIF1α), vascular endothelial growth factor (VEGF), and erythropoietin during phase 1, whereas these factors are upregulated during phase 2 (Ryu, 2022). Various signaling pathways, including HIF, VEGF, Wnt, Notch-Sox, and Semaphorin, are activated in ROP (Chen et al., 2011; Lee et al., 2014; Hartnett, 2015; Yang et al., 2015; Kim et al., 2016; Ramshekar and Hartnett, 2021).

Currently, laser photocoagulation has been used as a first-line therapy to treat ROP (Shulman and Hartnett, 2018; Linghu et al., 2022). Although laser therapy is a widely used method to treat ROP, it has been shown to increase the risk of myopia and other unfavorable ocular outcomes, such as macular dragging (Mintz-Hittner et al., 2011; Geloneck et al., 2014; Stahl et al., 2019). Anti-VEGF agents are the alternative therapy to treat ROP. Several anti-VEGF drugs including bevacizumab, ranibizumab, pegaptanib, and aflibercept have been investigated in the context of ROP, but only ranibizumab has received the indication for the treatment of ROP in Europe and Japan (Lee and Shirley, 2021). Although, long-term systemic effects of VEGF inhibition on other organs have been reported, there is no consensus on the optimal dosage and timing of anti-VEGF agent administration (Sato et al., 2012; Harder et al., 2014; Han et al., 2018; Hamad et al., 2020; Seery et al., 2020). Thus, it is worthwhile to investigate novel therapeutic targets to treat ROP and reveal more detailed mechanisms regulating ROP.

### Noncoding RNAs

Noncoding RNAs (ncRNAs) are RNAs that do not generally translate proteins. Various types of ncRNAs are reported to play a critical role in regulating heart, vascular, and neuronal diseases (Poller et al., 2018; Jae and Dimmeler, 2020; Ryu et al., 2021; Yoon et al., 2021; Ryu et al., 2022). Compared to messenger RNA (mRNA), the function of ncRNAs has not been thoroughly investigated. In this article, we will focus on the role of three different types of ncRNAs: microRNA (miRNA), long noncoding RNA (lncRNA), and circular RNA (circRNA).

miRNAs, relatively short ncRNAs with length around 22 nucleotides, have been widely studied in diverse diseases including ocular diseases. Various miRNAs were differentially expressed in vitreous humor of patients with ocular diseases (Ragusa et al., 2013). In addition, expression of miRNAs in vitreous humor of patients with uveal melanoma was analyzed and several miRNAs such as miR-146a were significantly upregulated suggesting their potential use as diagnostic biomarkers (Ragusa et al., 2015). miRNAs are highly conserved in mammals and function as modulators during post-transcription. They can bind to mRNA and inhibit protein translation or completely break down mRNA (Esteller, 2011). Single miRNAs can target multiple mRNAs and may involve in various biological processes (Hashimoto et al., 2013).

lncRNAs, longer-length ncRNAs, are typically longer than 200 nucleotides. Although lncRNAs have 5′ capped ends and are spliced showing similar characteristics as those of mRNAs, they lack open reading frames (ORF) resulting in the absence of protein-coding potential. Compared with mRNAs, the expression of lncRNAs is relatively weak and may differ depending on the types of species, tissue, or cell (Derrien et al., 2012). Moreover, the roles of lncRNAs may differ based on their localization. lncRNAs localized in the nucleus can modulate gene expression, whereas lncRNAs localized in the cytoplasm may function as miRNA sponges (Fatica and Bozzoni, 2014).

circRNAs, circular form RNA without 5′ and 3′ ends, are variable in length and are most frequently created by back-splicing events. Previously, circRNAs were considered as byproducts of mRNA processing; however, the diverse regulatory roles of circRNAs have been reported in various diseases. The most extensively studied function of circRNAs is their possible role as miRNA sponges (Hansen et al., 2013; Lasda and Parker, 2014). Additionally, circRNAs have been reported to act as protein sequesters or are involved in translation in cases where an ORF is included within the circRNAs. circRNAs are stable because they are not readily degraded by exonuclease due to their circular structure (Ryu et al., 2021). Thus, circRNAs may be a promising approach to treat various diseases. Recently, ncRNAs have also been investigated in various retinal vascular diseases. Compared to other retinal vascular diseases, the pharmacologic treatment options for ROP are limited. Thus, in this article, we review studies of ncRNAs in ROP and discuss their potential as novel therapeutic targets for the treatment of ROP.

## Noncoding RNA studies in retinopathy of prematurity

**FIGURE 1:**
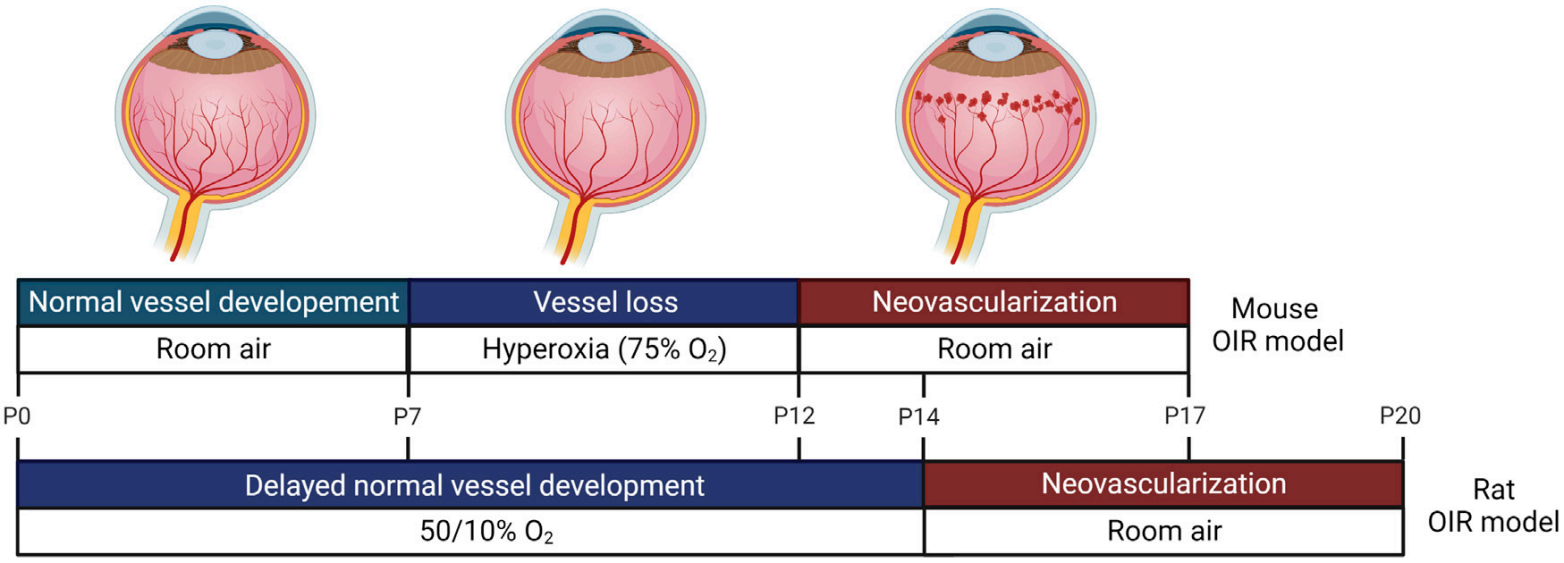
Oxygen-induced retinopathy (OIR) animal models. Animal models commonly used to investigate retinopathy of prematurity (ROP) are shown. In an OIR mice model, mouse pups and their mothers are placed in hyperoxia 7 days after birth for 5 days, which mimics phase 1 of ROP in humans. During hyperoxia, shrinkage of newly developed vessels occurs. The animals are then returned to room air (relative hypoxia) for 5 days, which resembles phase 2 of human ROP. During hypoxia, retinal neovascularization (RNV) begins and maximizes at 17 days after birth. In an OIR rat model, rat pups and their mothers are exposed to 50% oxygen on the first day and 10% oxygen on the next day, and this cycle is repeated for 14 days. During this fluctuating oxygen level period, normal vessel development is delayed. From 14 to 20 days, the rat pups are moved to room air, and RNV is induced.

**FIGURE 2:**
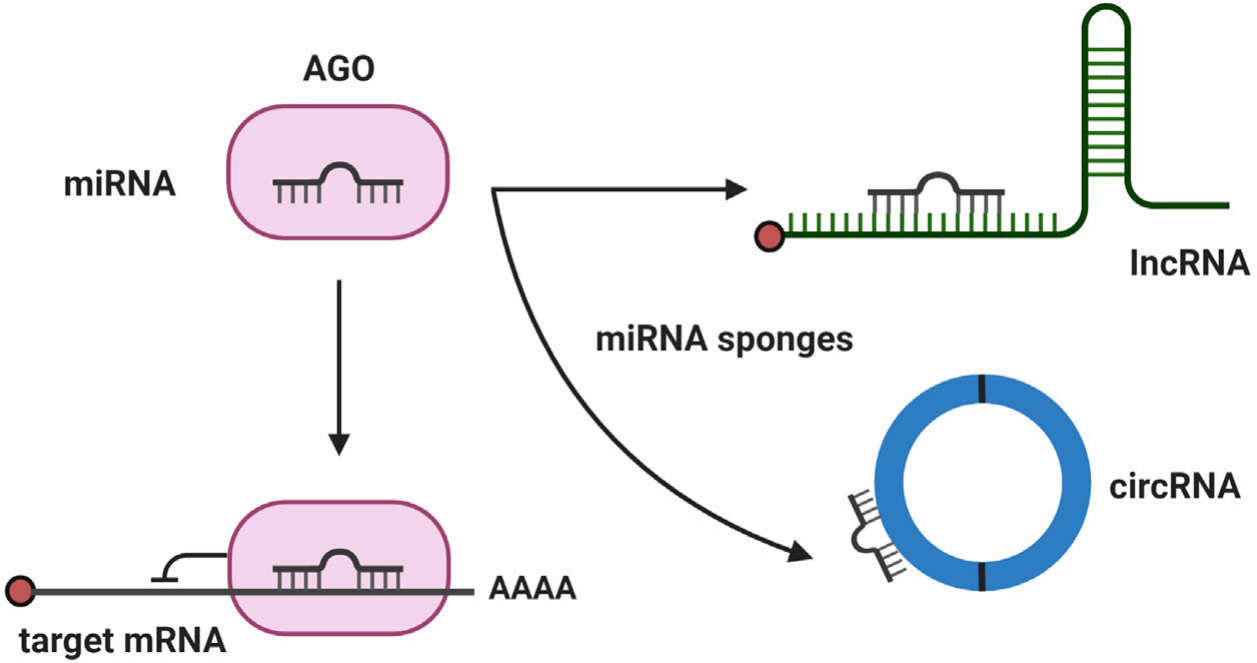
Major mechanism of noncoding RNAs (ncRNA). The major mechanisms of ncRNAs regulating ROP are depicted. microRNAs (miRNAs) bind with their target mRNA and degrade mRNA or inhibit translation. When long noncoding RNA or circular RNA act as a miRNA sponge, it can prevent miRNA binding with its target mRNA and recover mRNA processing.

We searched Pubmed and Embase databases for ncRNAs studies in the context of ROP, using the ncRNA-relevant search terms such as miRNA, lncRNA, and circRNA, and disease-related terms, such as retinopathy of prematurity, retinopathy, preterm infants, oxygen-induced retinopathy, retinal neovascularization, vaso-proliferation, vessel loss, and vaso-obliteration. We then manually reviewed the title and abstract of the articles to verify their relevance to our topic and selected articles for review (Tables 1, 2). By analyzing ncRNA studies in the context of ROP, we found that most studies focused on phase 2 of human ROP, in which retinal neovascularization (RNV) is maximized and the anti-angiogenic function of ncRNAs was investigated. Additionally, we found that the majority of studies were conducted in oxygen-induced retinopathy (OIR) *in vitro* or *in vivo* models. In the OIR *in vitro* model, human retinal microvascular endothelial cells (HRMECs), human retinal endothelial cells (HRECs), or human umbilical endothelial cells (HUVECs) were commonly used. Introducing cells to hyperoxia induced vaso-obliteration, whereas exposing cells to hypoxia promoted RNV. In addition, VEGF, H_2_O_2_, or CoCl_2_ was applied to cells to induce ROP. In the case of OIR *in vivo* models, mice or rats were commonly used, and the oxygen level was altered to mimic human ROP (Figure 1). miRNAs were the most commonly studied ncRNAs in ROP. Many miRNAs were found to target angiogenesis-related genes, such as VEGF, HIF1α, and Angiopoietin-2 (Figure 2). Compared to published research on miRNAs, there are comparatively few studies of lncRNAs and circRNAs in the context of ncRNAs in ROP. Most lncRNA and circRNAs were reported to function as miRNA sponges and indirectly regulate the target of miRNA (Figure 2).

**TABLE 1:** microRNAs regulating ROP.

miRNA	Expression	Study phase	Effects on ROP	Study model	Key findings	Reference
18a-5p	Up	Phase 2	Inhibits RNV	*in vivo*: OIR mice	Inhibits FGF1 and HIF1α	Guan et al. (2020)
34a	Down	Phase 2	Inhibits RNV	*in vitro*: VEGF-treated HRMECs *in vivo*: OIR rats	Inhibits Notch1	Shi et al. (2019)
96	Down	Phase 1	Inhibits vessel loss	*in vitro*: hyperoxia-induced HRMECs *in vivo*: OIR rats, vaso-obliteration model (80% O_2_) *ex vivo*: choroid isolated from rats at vaso-obliteration phase	Regulates VEGF and Angiopoietin-2	Desjarlais et al. (2020)
145	Up	Phase 2	Promotes RNV	*in vitro*: hypoxia-treated HRMECs *in vivo*: OIR mice	Inhibits TMOD3	Liu et al. (2019)
150	Down	Phase 2	Inhibits RNV	*in vitro*: VEGF-induced HRMECs *in vivo*: miR-150 KO mice *ex vivo*: aortic rings and choroidal explants from miR-150 KO mice	Inhibits CXCR4, DLL4, or FZD4	Liu et al. (2015)
181a-5p	Down	Phase 2	Inhibits RNV	*in vitro*: VEGF-induced HRECs *in vivo*: OIR mice	Inhibits Endocan	Chen et al. (2020)
182-5p	Down	Phase 2	Inhibits RNV	*in vitro*: hypoxia-induced HRMECs *in vivo*: OIR mice	Inhibits ANG and BDNF	Li et al. (2022)
299	Down	Phase 2	Inhibits RNV	*in vitro*: COCl_2_-induced HRECs *in vivo*: OIR mice	Inhibits VEGF-A	Wang et al. (2022)

ANG, angiogenin; BDNF, brain-derived neurotrophic factor; CXCR4, C-X-C chemokine receptor type 4; DLL4, delta like ligand 4; FGF1, fibroblast growth factor 1; FZD4, frizzled class receptor 4; HIF1a, hypoxia-inducible factor 1-alpha; HREC, human retinal endothelial cell; HRMEC, human retinal microvascular endothelial cells; KO, knockout; miRNA, microRNA; Notch1, neurogenic locus notch homolog protein 1; OIR, oxygen-induced retinopathy; RNV, retinal neovascularization; TMOD3, tropomodulin3; VEGF, vascular endothelial growth factor.

**TABLE 2:** long noncoding RNAs and circular RNAs regulating retinopathy of prematurity (ROP).

lncRNA	Expression	Study phase	Effects on ROP	Study model	Key findings	Reference
MALAT1	Up (P12)	Phase 2	Promotes RNV	*in vivo*: OIR mice	Inhibition of MALAT1 reduced RNV	Wang et al. (2020)
	Up	Phase 2	Promotes RNV	*in vitro*: hypoxia-induced HUVECs *in vivo*: OIR mice	Functions as miR-124-3p sponge and regulates EGR1 Inhibition of MALAT1 reduced RNV	Xia et al. (2021)
MEG3	Down (P12)	Phase 2	Inhibits RNV	*in vivo*: OIR mice	Overexpression of MEG3 inhibited RNV through PI3K/AKT/VEGF signaling pathway and reduced the expression of inflammatory factors	Di et al. (2022)
MIAT	Not applicable	Phase 2	Promotes RNV	*in vivo*: OIR mice	Inhibition of MIAT1 reduced RNV by regulating PI3K/AKT/VEGF signaling pathway	Di et al. (2021)
TUG1	Up	Phase 2	Promotes RNV	*in vitro*: CoCl_2_-treated HRECs *in vivo*: OIR mice	Acts as miR-299 sponge and regulates VEGF-A	Wang et al. (2022)
circRNA						
circPDE4B	Down	Phase 2	Inhibits RNV	*in vitro*: hypoxia-induced HRECs *in vivo*: OIR mice	Acts as miR-181c sponge and regulates VHL	Deng et al. (2020)
circZNF609	Up (P17)	Phase 2	Promotes RNV	*in vitro*: H_2_O_2_ or CoCl_2_-treated HUVECs *in vivo*: OIR mice	Functions as miR-615-5p sponge and modulates MEF2A Inhibition of circZNF609 reduced vessel loss and pathological RNV	Liu et al. (2017)
OIR retinal circRNAs	Up/down	Phase 2	Not applicable	*in vivo*: OIR mice	May work as ceRNAs in ROP	Zhou et al. (2019)

AKT, protein kinase B; ceRNA, competing endogenous RNA; circRNA, circular RNA; EGR1, early growth reponse 1; HREC, human retinal endothelial cell; HUVEC, human umbilical endothelial cells; lncRNA, long noncoding RNA; MALAT1, metastasis-associated lung adenocarcinoma transcript 1; MEF2A, myocyte Enhancer Factor 2A; MEG3, maternally expressed gene 3; MIAT, myocardial infarction-associated transcript; OIR, oxygen-induced retinopathy; PI3K, phosphoinositide 3-kinase; RNV, retinal neovascularization; ROP, retinopathy of prematurity; VEGF, vascular endothelial growth factor; VHL, von Hippel-Lindau.

### MicroRNAs regulating retinopathy of prematurity

Various miRNAs have been investigated for their effects on ROP (Table 1). The role of miR-18a-5p was studied in both *in vivo* and *in vitro* OIR model (Guan et al., 2020). miR-18a-5p was found to be upregulated in the OIR retina. Invitreal administration of the miR-18a-5p mimic, agomiR-18a-5p, significantly reduced the neovascular area in OIR mice retina. Similarly, agomiR-18a-5p-treated HRMECs showed reduced proliferation, migration, and tube formation. In addition, direct targets of miR-18a-5p, fibroblast growth factor 1 (FGF1) and HIF1α, were found. The mRNA expressions of FGF1 and HIF1α were significantly reduced after agomiR-18a-5p treatment in HRMECs. This study revealed that miR-18a-5p regulates pathological angiogenesis by targeting HIF1α and FGF1 in the OIR model.

The effect of miR-34a on retinal angiogenesis was studied in OIR *in vitro* and *in vivo* model (Shi et al., 2019). Previously, miR-34a was reported to inhibit tumor angiogenesis in endothelial cells, and the neurogenic locus notch homolog protein 1 (Notch1) pathway has been reported to play an important role in vascular endothelial growth factor (VEGF)-treated angiogenesis (Liu et al., 2003; Kumar et al., 2012). Therefore, the potential relationship of miR-34a and Notch1 in retinal angiogenesis was also investigated. miR-34a was downregulated, whereas Notch1 was upregulated in the *in vivo* OIR rat models. Similarly, administration of miR-34a mimic significantly reduced the expression of Notch1 in VEGF-induced HRMECs. Additionally, silencing of Notch1 significantly suppressed proliferation, migration, and tube formation in VEGF-induced HRMECs. Thus, it was shown that miR-34a reduces retinal neovascularization through inhibition of Notch1.

The role of miR-96 was investigated by Desjarlais et al. (2022). Expression of miR-96 was found to be downregulated in OIR rats and hyperoxia-induced HRMECs. Moreover, miR-96 mimics upregulated pro-angiogenic factors, such as VEGF, Angiopoietin-2, and FGF2, whereas antagomiR-96 inhibited these factors. Additionally, intravitreal injection of miR-96 mimic before hyperoxia significantly suppressed the vessel loss, which suggests that miR-96 has vaso-protective properties.

miR-145 was found to play a significant role in the regulation of endothelial cells during pathological angiogenesis (Liu et al., 2019). The investigators revealed that miR-145 directly targets tropomodulin3 (TMOD3), an actin-capping protein. The expression of miR-145 was upregulated, whereas the expression of TMOD3 was downregulated in the retinas of the OIR mice model and hypoxia-treated HRMECs. The function of miR-145 was investigated in an *in vitro* and *in vivo* OIR model. miR-145 inhibitor reduced RNV, whereas miR-145 mimic promoted retinal angiogenesis in HRMECs. Administration of miR-145 mimic reduced expression of TMOD3 and altered the structure of actin and endothelial cells. Additionally, either miR-145 inhibitor or miR-145 inhibitor along with small interfering RNA (siRNA) of TMOD3 was administered intravitreally in the eyes of OIR mice. Compared to the control group, the mice group injected with miR-145 inhibitor showed a significant reduction in neovascular area. However, when TMOD3 was suppressed along with miR-145, the neovascular region increased significantly. These results demonstrated that miR-145 regulates TMOD3 and revealed the role of the miR-145/TMOD3 axis in pathological RNV of the OIR model.

miR-150 was reported to involve in the pathological RNV (Liu et al., 2015). The authors found that expression of miR-150 was reduced in the retinal vessels of OIR mice. miR-150 mimic significantly inhibited proliferation, migration, and tube formation in HRMECs and the neovascular region in an OIR *in vivo* model. Targets of miR-150, C-X-C chemokine receptor type 4 (CXCR4), delta-like ligand 4 (DLL4), and frizzled class receptor 4 (FZD4) were identified by using the seed region sequence of miR-150. The miR-150 mimics were shown to inhibit the expression of CXCR4, DLL4, and FZD4 in HRMECs. Moreover, compared to the control, significant enlargement of the sprouting area of the aortic ring and choroid was detected in *ex vivo* experiments of miR-150 knockout mice. This study revealed the anti-angiogenic role of miR-150 in retinal neovascularization and downstream target genes of miR-150.

The role of miR-181a-5p in RNV was investigated in an OIR model (Chen et al., 2020). Previously, endocan was found to regulate the expression of proangiogenic factors, such as VEGF-A and VEGF-C, and be involved in cell activation and angiogenesis of endothelial cells. Endocan was upregulated in an *in vivo* experiment using OIR mouse model retinas. Suppression of endocan reduced survival, proliferation and tube formation in VEGF-treated HRECs and the neovascular area in OIR mice retinas. Additionally, the target of endocan, miR-181a-5p, was predicted using bioinformatics analysis and verified through the use of a luciferase assay. miR-181a-5p mimic inhibited proliferation, tube formation, survival in VEGF-treated HRECs. Moreover, overexpression of miR-181a-5p reduced the neovascular area in OIR mice retinas. Although the authors found the anti-angiogenic function of miR-181a-5p and miR-181a-5p/endocan regulatory axis, the effects of miR-181a-5p on other angiogenic pathways remain to be discovered in further research.

In a study by Li et al. (2022) the target of angiogenin (ANG) and brain-derived neurotrophic factor (BDNF) was revealed to be miR-182-5p. ANG was shown to be a pro-angiogenic factor that accelerates cell growth and endothelial tube formation (Miyake et al., 2015). Additionally, BDNF was found to promote migration and angiogenesis in endothelial cells (Matsuda et al., 2012). Through bioinformatics, the authors predicted that miR-182-5p was a potential target of ANG and BDNF. The expression level of miR-182-5p was downregulated, whereas the expressions of ANG and BDNF mRNA were upregulated in the retinas of OIR mice and hypoxia-induced HRMECs. In addition, when miR-182-5p mimic was introduced, the expression of ANG and BDNF was reduced in hypoxia-induced HRMECs. Compared to the scramble group, the miR-182-5p mimic group showed decreased cell migration and increased cell viability and tube formation in hypoxia-induced HRMECs (Li et al., 2022). Thus, the authors discovered that miR-182-5p, ANG, and BDNF can be potential targets to treat RNV.

### Long noncoding RNAs regulating retinopathy of prematurity

Several lncRNAs have been investigated in terms of their role in ROP (Table 2). The pro-angiogenic role of lncRNA metastasis-associated lung adenocarcinoma transcript 1 (MALAT1) was revealed in two studies (Wang et al., 2020; Xia et al., 2021). Wang et al. reported that lncRNA MALAT1 expression was upregulated in OIR mice. Compared to the control, inhibition of MALAT1 reduced RNV and suppressed CCN1/Akt/VEGF pathway and inflammatory cytokines, including IL-1β, IL-6, and TNF-α, during hyperoxia (Wang et al., 2020). These results suggest that the inhibition of lncRNA MALAT1 may reduce the progression of ROP. In addition, Xia et al. revealed that lncRNA MALAT1 can act as an miR-124-3p sponge and modulate early growth response 1 (EGR1) (Xia et al., 2021). The expression levels of miRNAs, lncRNAs, and mRNAs in an OIR mice model were evaluated in the microarray. miR-124-3p, a significantly downregulated miRNA in microarray, was selected for further study. As shown in the microarray, expression of miR-124-3p expression was significantly reduced in a hypoxia-induced *in vitro* model. The addition of miR-124-3p inhibited proliferation and migration, whereas suppression of miR-124-3p promoted proliferation and migration of hypoxia-treated HUVECs. Through bioinformatics analysis, the interacting partners of miR-124-3p, lncRNA MALAT1, and EGR1 were predicted. The expression of EGR1 and lncRNA MALAT1 was upregulated in hypoxia-treated HUVECs and retinas of OIR mice. Overexpression of miR-124-3p or inhibition of MALAT1 also suppressed EGR1 in hypoxia-induced HUVECs. Thus, the results of the study revealed the novel regulatory axis of lncRNA MALAT1/miR-124-3p/EGR1 in OIR *in vitro* and *in vivo* models.

The role of the maternally expressed gene 3 (MEG3) in ROP was revealed by Di et al. (2022). Intravitreal injection of MEG3 overexpressing lentivirus reduced retinal angiogenesis *via* VEGF/phosphoinositide 3-kinase (PI3K)/protein kinase B (Akt) signaling pathway and suppressed inflammatory markers, such as IL-1β and IL-6 in OIR mice. Additionally, this research group also investigated the effect of lncRNA myocardial infarction-associated transcript (MIAT) in the OIR mice model (Di et al., 2021). The investigators found that silencing lncRNA MIAT by administering intravitreal injection suppressed retinal angiogenesis through downregulation of the VEGF/PI3K/Akt pathway. Thus, lncRNA MEG3 or MIAT may be a promising therapeutic target to treat ROP.

Lastly, Wang et al. explored the function of lncRNA TUG1 in retinal angiogenesis (Wang et al., 2022). Previously, lncRNA TUG1 was studied in various cancer; however, its role in retinal angiogenesis was not investigated. The authors found that the expression of TUG1 was upregulated in the retinas of OIR mice, whereas the expression of miR-299-3p was downregulated. They showed that knockdown of lncRNA TUG1 reduced RNV, apoptosis, inflammation, and the level of angiogenesis markers such as VEGF-A in OIR mice retinas. Moreover, lncRNA TUG1 was found to act as a miR-299-3p sponge and modulate VEGF-A. Overexpression of miR-299 inhibited VEGF and TUG1 and reduced tube formation, migration, and apoptosis in CoCl_2_-treated HRECs.

### Circular RNAs regulating retinopathy of prematurity

There are a few studies in which the role of circRNA in ROP has been investigated (Table 2) (Liu et al., 2017; Zhou et al., 2019; Deng et al., 2020). Deng et al. reported that the expression of circPDE4B was decreased in hypoxia-treated HRECs and retinas of OIR mice (Deng et al., 2020). Overexpression of circPDE4B inhibited the expression of angiogenic factors, such as HIF1α and VEGF-A, cell proliferation, and vascular tube formation *in vitro*. Moreover, the authors found that circPDE4B works as a miR-181c sponge and modulates von Hippel-Lindau (VHL). This study revealed the anti-angiogenic function of circPDE4B and found that circPDE4B/miR-181c/VHL regulatory axis regulates ROP.

Liu et al. investigated the function of circZNF609 (Liu et al., 2017). The expression of circZNF609 was upregulated during hypoxia*.* Inhibition of circZNF609 promoted cell viability, migration, and tube formation and suppressed RNV in OIR mice model. Using bioinformatics databases, the investigators predicted miR-615-5p would interact with circZNF609. CircZNF609 was verified to act as a miR-615-5p sponge in H_2_O_2_-treated HUVECs. Subsequently, the downstream target of miR-615-5p, Myocyte Enhancer Factor 2A (MEF2A), was predicted using a bioinformatic database. Overexpression of MEF2A reduced cell migration and tube formation promoted by inhibition of circZNF609. Therefore, circZNF609/miR-615-5p/MEF2A axis was revealed to regulate vascular endothelial cell function.

CircRNA profiles of retinas from OIR and normal mice were analyzed by Zhou et al., 2019. They revealed differentially expressed circRNA, miRNA, and mRNA in the OIR mice model. Based on gene ontology analysis, angiogenesis was found to be one of the more prevalent biological processes. The potential of circRNA acting as competing endogenous RNA (ceRNA) was predicted using a bioinformatics database, miRanda. The levels of expression of selected circRNA, miRNA, and mRNAs were verified using RT-qPCR, suggesting that various circRNA-miRNA-mRNA regulatory axes may be involved in the progression of ROP.

## Strategies to modulate ncRNAs

By overexpressing or inhibiting ncRNAs, the progression and severity of ROP can be modulated. miRNA can be overexpressed by using miRNA mimics or microRNA expression vectors. miRNA mimic, a synthetic miRNA with the identical sequence as an endogenous miRNA, can be used to upregulate expression of miRNA. Double-stranded miRNA mimic is processed to single-stranded miRNA inside RNA-induced silencing complex (RISC) and subsequently inhibit target mRNA (van Rooij and Kauppinen, 2014). Several miRNA mimics have been investigated in clinical trials. For example, MRX34, the miR-34a mimic, was investigated in phase 1 clinical trials with liver cancer (Beg et al., 2017; Hong et al., 2020). miRNA expression vectors are promoter-containing vectors designed to express miRNAs of interest (Ling et al., 2013). For instance, miR-26a expression vector was used to inhibit the progression of cancer in the *in vitro* and *in vivo* hepatocellular carcinoma model (Kota et al., 2009). On the other hand, miRNAs can be suppressed by anti-miR or miRNA sponge (Ling et al., 2013). Anti-miR is an antisense oligonucleotide that inhibit target miRNAs and has a partially or fully complementary sequence to its target endogenous miRNA. Miravirsen, miR-122 anti-miR, was tested for the treatment of hepatitis C virus infection in phase 2 clinical trial (Janssen et al., 2013). miRNA sponge vectors are designed to contain multiple complementary sequence sites of single or multiple miRNAs of interest (Ebert et al., 2007; Chang, 2018). For instance, the miR-122 sponge vector reduced miR-122 in liver and effectively inhibited cholesterol level for 25 weeks in miR-122 sponge vector-injected mice (Xie et al., 2012). Furthermore, backbone or sugars of miRNA mimics or miRNA inhibitors can be modified to enhance stability (Baumann and Winkler, 2014).

LncRNAs can be overexpressed by constructing lncRNA overexpression plasmids. For instance, promoter region of lncRNAs can be combined with CRISPR activator complex to upregulate transcription of lncRNA (Dominguez et al., 2016; Lim et al., 2020). Strategies to inhibit lncRNA vary depending on localization. Nuclear lncRNAs can be suppressed by antisense oligonucleotides *via* degradation through RNase H. On the other hand, cytoplasmic lncRNAs can be suppressed by siRNAs *via* RNA interference (Lennox and Behlke, 2016). In addition, CRISPR-Cas 13 system can be used to inhibit or degrade lncRNA (Cox et al., 2017; Zhang et al., 2020). CircRNA can be upregulated using the circRNA overexpression vector through induction of backsplicing (Kramer et al., 2015). On the contrary, circRNAs can be downregulated by siRNAs, given that most circRNAs are enriched in the cytoplasm (Jeck et al., 2013).

## Future perspective on ncRNA therapy

Recently, RNA therapies have received attention, and several ncRNA therapeutics are under clinical trials (Huang et al., 2020; Winkle et al., 2021). Various ncRNAs have been investigated for their uses in diagnosis and treatment. To date, miRNA therapeutics have been explored in various diseases, including hepatitis C virus infection and cancer, and have undergone clinical trials (Janssen et al., 2013; Beg et al., 2017; van der Ree et al., 2017). Currently, there are few pharmacological options for the treatment of ROP, and laser therapy can be invasive and increase the risk of myopia and other undesirable ocular outcomes; thus, it is worth finding novel therapeutic targets (Ryu, 2022). Compared to conventional therapy, ncRNA therapy can be effective against targets that have been unresponsive to the currently available drugs and the stability of RNA therapy can be enhanced by using carriers, such as liposomes (Ozpolat et al., 2014; Damase et al., 2021). Additionally, RNA therapy does not cause gene alteration. Because ROP occurs in preterm infants, the incidence is low compared to other retinal diseases such as diabetic retinopathy, it may not be a promising research and development target for pharmaceutical companies. Thus, RNA therapy is a reasonable treatment option in ROP, especially given that it is less expensive to develop than conventional therapy.

## Conclusion

Although laser therapy and anti-VEGF agents have been used to treat ROP, the ROP incidence has increased and current therapies pose risks due to invasive methods and lack of data on long-term safety issues and dosage. Recently, RNA therapy has been extensively investigated in diverse diseases and has shown potential as a novel therapy. Among the different types of RNAs, ncRNAs have been investigated as emerging therapeutics in many diseases. In this article, we discussed the role of ncRNAs, including miRNAs, lncRNAs, and circRNAs, that have been investigated in the context of ROP. Because ROP can be regulated through overexpression or inhibition of ncRNAs, modulation of ncRNA can be a novel therapeutic approach to treat ROP.
